# Autophagy in the retinal neurovascular unit: New perspectives into diabetic retinopathy

**DOI:** 10.1111/1753-0407.13373

**Published:** 2023-03-02

**Authors:** Xiongyi Yang, Zexin Huang, Mei Xu, Yanxia Chen, Mingzhe Cao, Guoguo Yi, Min Fu

**Affiliations:** ^1^ Zhujiang Hospital Southern Medical University Guangzhou Guangdong P. R. China; ^2^ The Second Clinical School Southern Medical University Guangzhou Guangdong P. R. China; ^3^ The Second People's Hospital of Jingmen Jingmen Hubei People's Republic of China; ^4^ Department of Ophthalmology, Zhujiang Hospital Southern Medical University Guangzhou Guangdong P. R. China; ^5^ Department of Ophthalmology, The Seventh Affiliated Hospital Sun Yat‐Sen University Shenzhen P. R. China; ^6^ Department of Ophthalmology, The Sixth Affiliated Hospital Sun Yat‐Sen University Guangzhou Guangdong P. R. China

**Keywords:** autophagy, diabetic retinopathy, mitophagy, retinal neurovascular unit, 自噬, 糖尿病性视网膜病变, 线粒体自噬, 视网膜神经血管单元

## Abstract

Diabetic retinopathy (DR) is one of the most prevalent retinal disorders worldwide, and it is a major cause of vision impairment in individuals of productive age. Research has demonstrated the significance of autophagy in DR, which is a critical intracellular homeostasis mechanism required for the destruction and recovery of cytoplasmic components. Autophagy maintains the physiological function of senescent and impaired organelles under stress situations, thereby regulating cell fate via various signals. As the retina's functional and fundamental unit, the retinal neurovascular unit (NVU) is critical in keeping the retinal environment's stability and supporting the needs of retinal metabolism. However, autophagy is essential for the normal NVU structure and function. We discuss the strong association between DR and autophagy in this review, as well as the many kinds of autophagy and its crucial physiological activities in the retina. By evaluating the pathological changes of retinal NVU in DR and the latest advancements in the molecular mechanisms of autophagy that may be involved in the pathophysiology of DR in NVU, we seek to propose new ideas and methods for the prevention and treatment of DR.

## Graphical abstract

Highlights

Autophagy is closely related to retinal neurovascular units in diabetes retinopathy. Different cells participate in the occurrence and development of diseases through autophagy, and targeted autophagy may be a new direction of treatment in the future.

- Emphasized the close relationship between diabetic retinopathy (DR) and autophagy
- Summarized the autophagy molecular mechanism of DR pathophysiology in the neurovascular unit
- Proposed potential targets for DR treatment

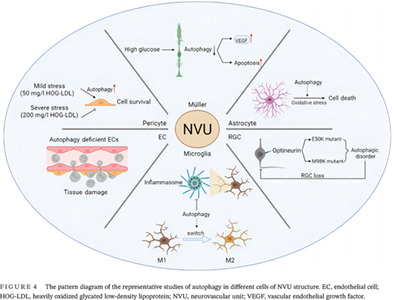

## 摘要

糖尿病视网膜病变(DR)是世界范围内最常见的视网膜疾病之一，是育龄人群视力损害的主要原因。研究表明，自噬是破坏和恢复胞质成分所需的一种重要的细胞内稳态机制，在DR中具有重要意义。自噬维持应激状态下衰老和受损细胞器的生理功能，从而通过多种信号调节细胞寿命。视网膜神经血管单元(NVU)作为视网膜的基本功能单位，在维持视网膜环境稳定和支持视网膜代谢需求方面起着至关重要的作用。然而，自噬对于NVU的正常结构和功能至关重要。本文就DR与自噬的密切关系、自噬的种类及其在视网膜中的重要生理活性进行综述。我们期望通过评估DR中视网膜NVU的病理变化以及自噬可能参与NVU中DR病理生理分子机制的最新进展，寻求防治DR的新思路和方法。

## 1 INTRODUCTION

Diabetes mellitus is a prevalent metabolic disorder that can result in numerous systemic complications.^1^ As a microvascular complication of diabetes, diabetic retinopathy (DR) is one of the leading causes of blindness worldwide.^2^ Some studies predict that the number of diabetics will reach 552 million by 2030, with over one third of patients showing signs of DR.^3^ Prolonged hyperglycemia can cause retinal microvascular basement membrane thickening, vascular cell loss, increased vascular permeability, and neovascularization.

The retina is made up of 10 layers: from outside, the retinal pigment epithelium (RPE), the rod and cone layer, the outer limiting layer, the outer nuclear layer (ONL), the outer plexiform layer (OPL), the inner nuclear layer (INL), the inner plexiform layer (IPL), the ganglion cell layer (GCL), the nerve fiber layer (NFL), and the inner limiting membrane.^4^ Neurons, glial cells, and blood cells in the retina are histologically connected to form a critical structure known as the retinal neurovascular unit (NVU).^5^ The retinal NVU is made up of ganglion cells, glial cells (astrocytes and Müller cells), immune cells (microglia), and vascular cells (endothelial and pericytes)^6^ demonstrating the intricate functional coupling and interdependence of different retinal cells. Increasing numbers of studies have linked retinal microangiopathy, retinal neurodegeneration, and inflammation to the pathophysiology of DR.^7^

Recent study has implicated autophagy in the pathogenesis of DR and has been linked to the progression and remission of DR.^8^ Autophagosomes in cells consume proteins, lipids, and even entire organelles before transporting them to lysosomes for elimination, which is an effective way to promote cell component degradation and circulation catabolism. In addition, autophagy not only plays a vital role in cell quality control, but it also plays a regulatory role in the degradation of cellular components in response to pressure, which is recycled to generate nutrients needed to maintain intracellular homeostasis.

Retinal NVU cells have been discovered to be able to mediate autophagy and actively participate in the pathogenesis of DR, making them an important disease‐related research object. However, current research lacks a systematic summary and is dispersed. Aiming to propose novel ideas and methods for preventing and treating DR, we reviewed autophagy's physiological functions in the retina, focusing on the pathological changes of retinal NVU in DR and the most recent advancements in autophagy in NVU.

## 2 MATERIALS AND METHODS

We found a total of 76 relevant articles by searching PubMed for the terms “retinal neurovascular unit” and “diabetic retinopathy” up to July 2022. Only English‐language papers were considered. The figures were created with BioRender.com.

## 3 AUTOPHAGY

### 3.1 Types of autophagy

**FIGURE 1:**
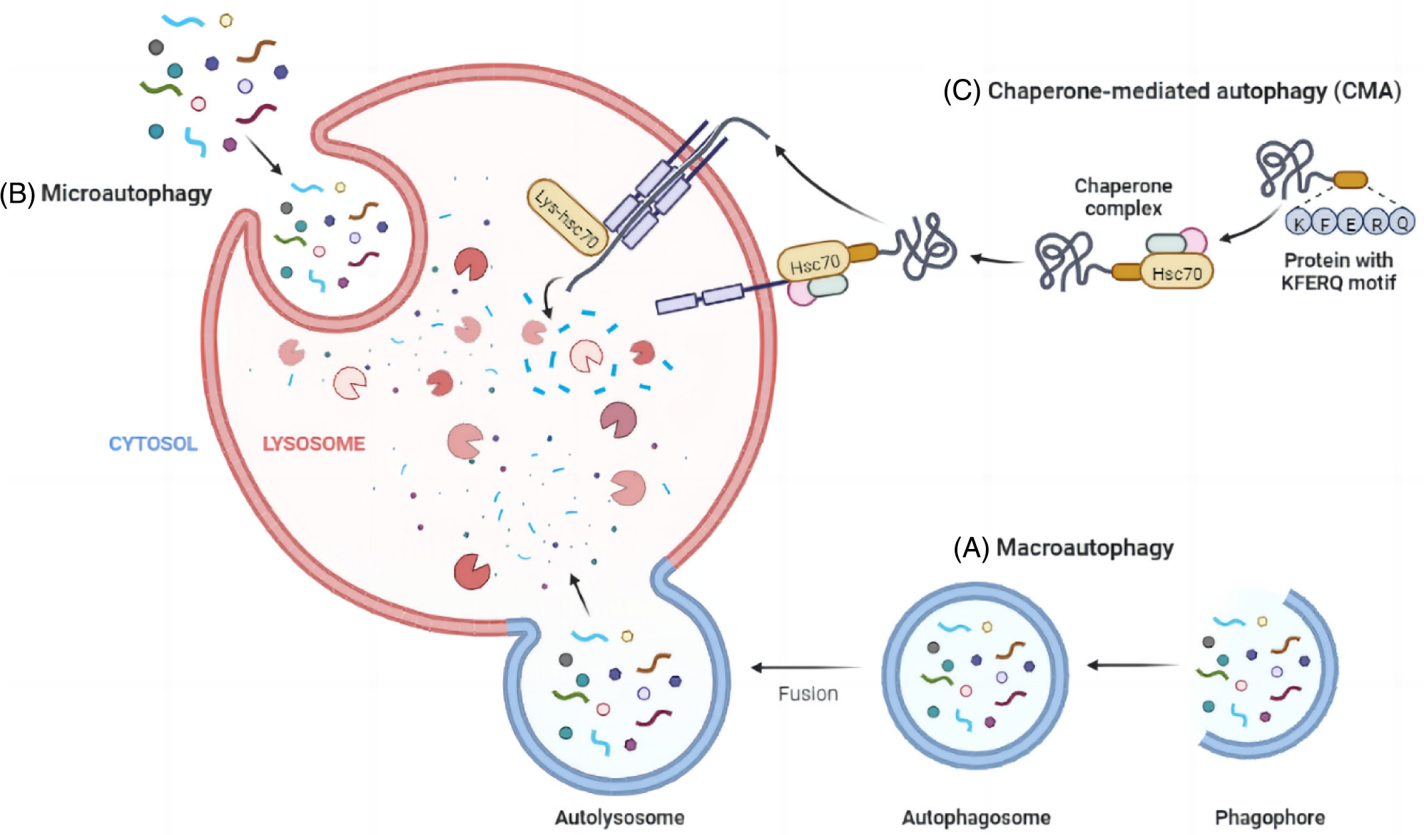
The main autophagy pathways described in mammalian cells are classified according to the way goods are delivered to lysosomes. (A), Macroautophagy promotes the circulation of intracellular components (including organelles) by forming autophagosomes. (B), In the process of microautophagy, proteins translocate directly through the invagination of lysosomal membrane. (C), Chaperone‐mediated autophagy can degrade proteins that carry a specific sequence (KFERQ motif), which is recognized by chaperone proteins such as Hsc70. The recognition of chaperone proteins by lysosomal‐associated membrane protein 2A (chaperone‐mediated autophagy receptor in lysosomal membrane) promotes the expansion and translocation of proteins in the lysosome.

The autophagy‐lysosome system allows cells to destroy their own components to maintain the balance between anabolism and catabolism.^9^ The autophagy process is strictly regulated by mammalian target of rapamycin (mTOR) and AMP‐activated protein kinase (AMPK)‐mediated signal pathways,^9^ but factors and conditions such as reactive oxygen species (ROS) and hypoxia can also promote autophagy function upregulation to maintain intracellular stability.^10^ Autophagy is classified into three types: macroautophagy, chaperone‐mediated autophagy (CMA), and microautophagy (Figure 1).

In macroautophagy, part of the cytoplasm is swallowed by the isolation membrane to form double‐membrane organelles called autophagosomes. The molecular mechanism of its formation has been elucidated: kinase ULK1 forms a complex with ATG13, ATG101, and FIP200, and ULK1 triggers the formation of PI3K complex composed of Vps34, Beclin‐1, Vps15, and Vps14. Next, ATG7 mediates the binding of ATG12, ATG5, and ATG16L, and also promotes the binding of phosphatidylethanolamine to light chain 3 protein (LC3) to form autophagosome binding LC3 (LC3‐II). Two ubiquitin binding systems are activated to complete the formation of autophagosomes. After the autophagosome binds to the lysosome, the substance in the cytoplasm is degraded with the help of the lysosome. It is worth noting that the whole process of macroautophagy is regulated by the ATG protein family. CMA was found to exist in mammalian cells. Lysosomal‐associated membrane protein can act on lysosomal receptors and mediate unfolded peptides to cross the lysosomal membrane into lysosomes and be degraded in lysosomes. Hsc70, a helper on the cell membrane, promotes this process. The third is microautophagy. Unlike macroautophagy, autophagosomes are not formed in the process of microautophagy, but rely on the action of lysosome membrane to directly absorb the components in the cytoplasm to achieve the purpose of clearance.

In addition to nonselective autophagy, selective autophagy has recently gained popularity.^9^ Selective autophagy refers to the process by which autophagosomes encircle a specific substrate and combine with lysosomes to degrade the substrate. According to the cargo type, selective autophagy is classified into numerous subtypes, such as mitophagy, endoplasmic reticulum (ER)‐phagy, and nucleophagy, among others.^11^ Both the maintenance of cellular homeostasis and the onset of disease are dependent on selective autophagy, which plays a role in the quality control of organelles and the response to supply of nutrients.^12^ Cancer, neurodegenerative disease, metabolic disease, and inflammatory disease can all be caused by defects in selective autophagy.^13^, ^14^ Consequently, regulating selective autophagy under pathological conditions may be crucial for disease treatment. This review focuses primarily on the effects of macroautophagy and mitophagy.

### 3.2 Physiological functions of autophagy in the retina

#### 3.2.1 Autophagy and quality control

**FIGURE 2:**
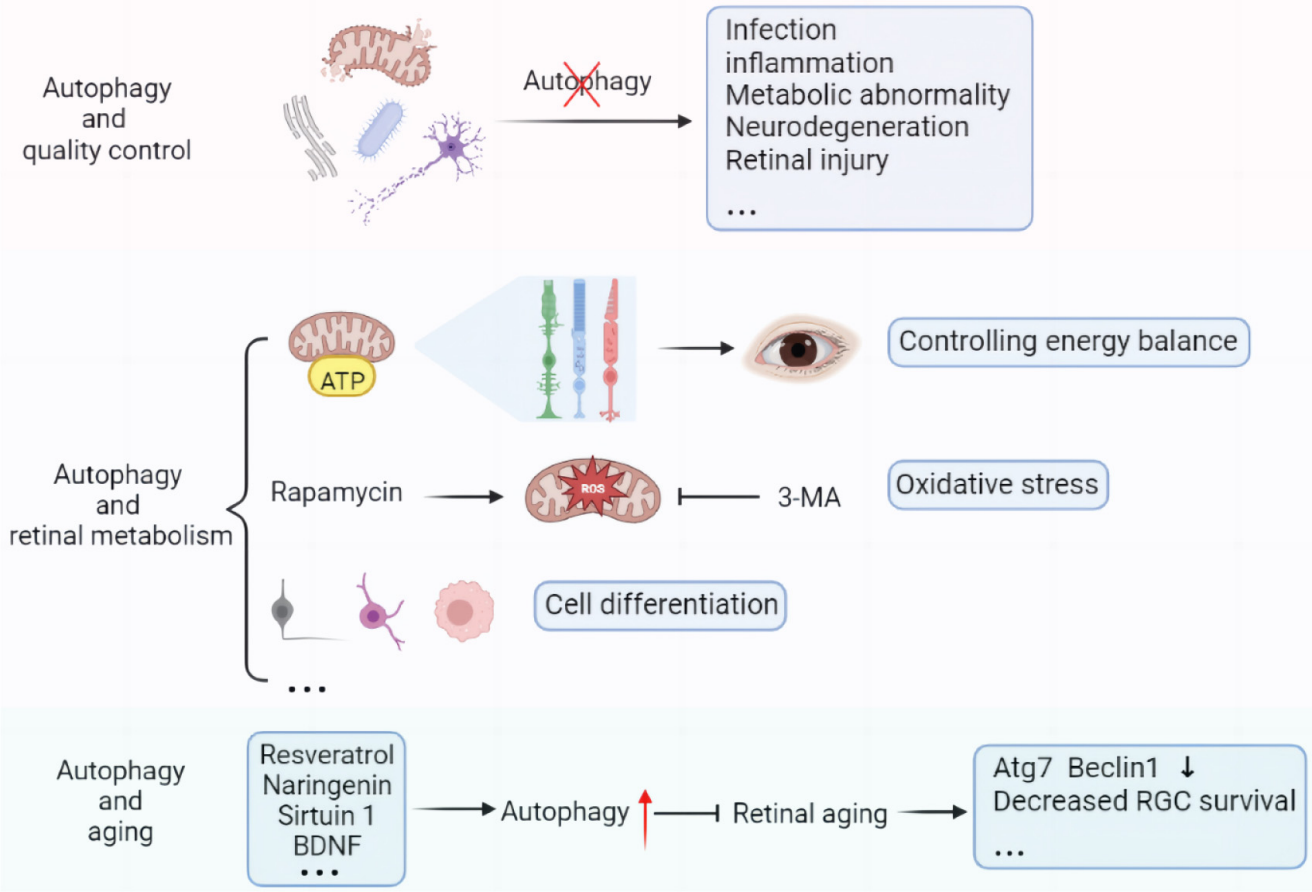
Main physiological functions of autophagy in the retina. Autophagy is one of the main cell quality control systems of proteins and organelles and is a metabolic process that can control the energy balance and then control the energy dynamic balance of a single cell and the whole organism. Autophagy is closely related to retinal senescence and the autophagy disorder is one of the mechanisms of retinal degeneration and loss of retinal cells. BDNF, brain‐derived neurotrophic factor; RGC, retinal ganglion cell; 3‐MA, 3‐methyladenine.

The ubiquitin‐proteasome system and autophagy are the primary systems for controlling protein and organelle quality in cells. They collaborate to ensure cell health and appropriate damage response.^15^ In neurodegenerative diseases,^15^ infections, inflammatory diseases,^16^, ^17^ metabolic diseases^18^ and other diseases, abnormal and defective proteins and organelles will accumulate in the case of autophagy dysfunction, thereby reducing the capacity of cells to resist and eliminate infectious pathogens. The same holds true for cancer. In the late stages of tumor progression, autophagy may mediate tumor promotion and development, but it can also maintain genome stability in the early stages, prevent chronic tissue damage and inflammation, and subsequently prevent tumor proliferation, invasion, and metastasis^19^ (Figure 2).

It has been demonstrated that autophagy maintains the dynamic balance of multiple postmitotic cells, such as neurons and hepatocytes. In the absence of neuronal autophagy, tubular endoplasmic reticulum accumulates selectively in axons, which causes an increase in excitatory neurotransmitters and the impairment of mouse vitality, according to a study of ATG5‐deficient knockout mice.^19^ Significant retinal changes resulted from selective deletion of ATG5 and ATG7 from mouse neuronal precursors, including autophagic substrate p62 reduction, ubiquitin accumulation in the entire retina, and cell death of photoreceptor.^20^ These findings suggest that autophagic cell quality control is critical for maintaining normal retinal physiological function.

#### 3.2.2 Autophagy and retinal metabolism

Autophagy is a metabolic pathway that regulates energy homeostasis and consequently the dynamic energy balance of a single cell and the entire organism (Figure 2). The Warburg effect demonstrates that the human retina primarily metabolizes most glucose via anaerobic glycolysis, producing lactic acid rather than adenosine triphosphate (ATP) via oxidative phosphorylation.^20^ Although the Warburg effect has received a lot of attention in the context of cancer research,^21^ it can also play an important role in providing a fast ATP supply for synaptic activities necessary for light transduction in the retina. In addition, Müller cells, the retina's primary glycogen storage cells, depend on aerobic glycolysis and lactic acid production.^22^ Interestingly, it was previously thought that the retina used glucose only as an energy substrate, but it has recently been proposed that fatty acid oxidation also contributes to the neuroretina's energy supply.^22^

Autophagy protects cells by regulating metabolic activity. With an active metabolism and an abundance of mitochondria, retinal RPE cells are vulnerable to oxidative damage, which significantly contributes to retinal degeneration. In studies of oxidative stress, the autophagy agonist rapamycin increases autophagy, reducing ROS production in RPE cells, whereas mitochondrial activity decreases significantly when autophagy is inhibited with ATG7/BECN1 gene knockout or 3‐methyladenine (3‐MA).^23^ When 3‐MA was applied to the developing chick retina, similar results were observed. The situation was improved by administering methyl pyruvate, a cell‐permeable substrate for ATP synthesis, to the retina.^24^ Autophagy is interestingly involved in the differentiation of numerous cell types. We found that neurogenesis of retinal ganglion cells (RGC) is controlled by programmed mitophagy. In addition to increasing the expression of mitochondrial autophagic receptor BNIP3L/NIX, the high stability of hypoxia inducible factor (HIF1A) also increases glycolysis. The integration of mitophagy and glycolysis accelerates the differentiation of RGC. This metabolic shift, dependent on mitophagy during M1 macrophage polarization, was also observed by Lorena et al.^25^ In addition, BNIP3L‐mediated mitophagy also regulates the oligodendrocyte differentiation.^26^

#### 3.2.3 Autophagy and retinal aging

Age‐related vision loss is thought to result from retinal degeneration and cell loss, with autophagy dysregulation being one of the underlying mechanisms^27^ (Figure 2). Reduced transcription of autophagy regulators like Atg7 and Beclin1 demonstrates that autophagy levels decline with age.^20^ Glaucoma, a prevalent age‐related disease, can cause progressive RGC death and vision loss. In the optic nerve crush model, aged autophagy‐deficient mice have significantly decreased RGC survival and altered oxidative stress response pathways as well as mitochondria.^28^ Similarly, RPE cells in age‐related macular degeneration (AMD) from double knockout mice of NFE2L2 and PGC1α,^29^ the master transcription factors regulating antioxidant enzymes and mitochondrial biogenesis, have reduced mitophagy and altered or absent cellular morphology and function.^30^ These findings imply that maintaining a certain level of autophagic activity in the eye can protect degenerating retinal cells.

Many drugs have been discovered to significantly ameliorate these adverse effects of retinal aging. The electroretinogram has been utilized to evaluate retinal function; a‐waves reveal details about photoreceptors, and b‐waves reveal details about the physiology of bipolar cells and Müller cells.^31^ Recent research demonstrates that naringenin treatment not only increases cone survival but also protects the retina by enhancing b‐wave amplitude.^32^ This protective mechanism, according to Chen et al, may be associated with naringenin's ability to promote autophagy initiation via mTOR signaling pathway and improve mitochondrial dynamics mediate.^33^ Furthermore, Sirtuin1, a gene involved in retinal degeneration, has been shown to mediate cardiomyocyte autophagy during glucose deprivation.^34^ There was a concurrent increase in sirtuin1 expression and b‐wave amplitude after 19 months of treatment for aged rats with resveratrol, an antioxidant and sirtuin1 activator, demonstrating attenuated retinal degeneration.^35^ Interestingly, Gastón Repossi et al discovered that the levels of brain‐derived neurotrophic factor (BDNF) and the neurotrophin receptor TrkB are boosted after resveratrol treatment,^36^ implying that retinal cell survival in this setting may be mediated by Sirtuin1, BDNF, and TrkB. Resveratrol, on the other hand, not only increases mitochondrial mass and function in the zebrafish retina but also inhibits Akt/mTOR activity in the aging retina, resulting in antiaging and neuroprotection.^37^

### 3.3 Autophagy in DR

DR is the result of a number of pathological complications caused by hyperglycemia and insulin signal pathway defects, which lead to retinal microvascular defects as well as neuroretinal dysfunction and degeneration.^2^ Although the molecular mechanism of DR has not yet been fully elucidated, mounting evidence suggests that oxidative stress, hypoxia, endoplasmic reticulum stress, and other DR‐related factors are all linked to autophagy.^38^ When treated with high glucose medium, endothelial cells and pericytes showed increased oxidative stress, as did other nonvascular retinal cells such as photoreceptors and Müller cells.^39^ Dysfunction in mitochondria is commonly attributed to oxidative stress within cells,^40^ which can be caused by an increase in ROS levels in DR or a decrease in the retina's ability to cope with oxidative stress, and this phenomenon has been confirmed in diabetes rats.^41^ Mitophagy appears to have anti‐inflammatory effects by blocking inflammasome activation outside of its scheduled time window,^42^ which is critical in preventing DR progression.

Furthermore, damage to RPE cells and photoreceptors has been shown to hasten the progression of DR. Tumor necrosis factor alpha (TNF‐α) released by Müller cells can aggravate RPE cell apoptosis by increasing mitophagy.^43^ Downregulation of high mobility group box 1 (HMGB1) expression has been shown to rescue lysosome membrane permeabilization, restore autophagy's degradation ability, reduce the expression of vascular endothelial growth factor (VEGF) and inflammatory factors, and protect RPE cells from apoptosis in early DR.^38^ On the subject of inflammation, researchers discovered that glial cell maturation factor β could induce ferroptosis in early DR by blocking degradation of ACSL4, a protein related to chaperone‐mediated autophagy,^44^ whereas circFAT1 can promote RPE cell autophagy and inhibit high glucose‐induced pyroptosis.^45^ Furthermore, inhibiting ARPE‐18 cell autophagy by 3‐MA causes the production of ROS, activating the nucleotide‐binding oligomerization domain, leucine rich repeat and pyrin domain containing (NLRP3) inflammasome, thereby leading to interleukin‐1β (IL‐1β) secretion.^46^ By regulating function of autophagy and mitochondrial, AMPK activation has been shown in recent studies to delay degeneration of photoreceptor caused by diabetic.^47^ Thioredoxin upregulation has also shown similar outcomes via this pathway.^48^ Under hyperglycemic conditions, autophagy may be more important in 661 W cells (a transformed murine cone cell line), and superoxide formation and apoptosis will be increased when inhibiting autophagy.^49^

The cellular and molecular connections between autophagy and DR are far from limited to these, but it is undeniable that autophagy is crucial in many aspects of DR pathogenesis and treating the retina against autophagy is critical.

## 4 THE RETINAL NVU IN DR

### 4.1 The retinal NVU

**FIGURE 3:**
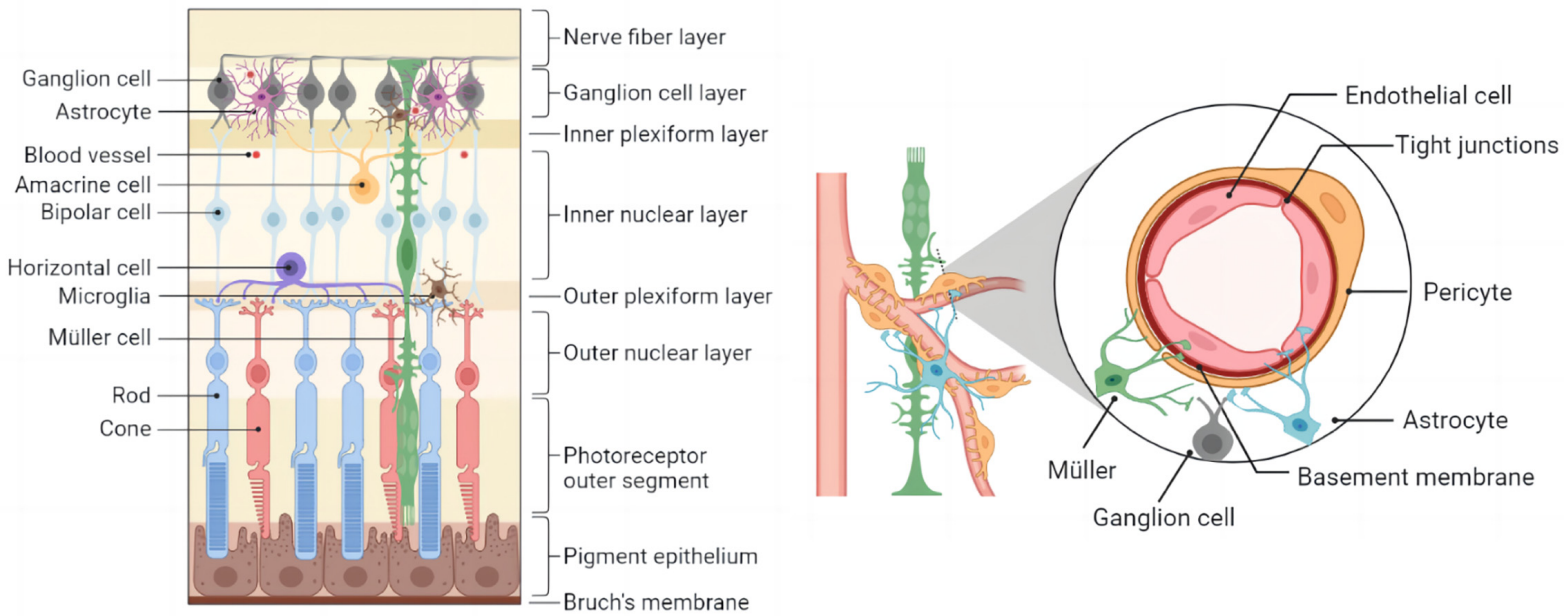
The retina is a tissue composed of several layers of cells. Retinal pigment epithelium is a layer of cells, which is in close contact with the outer layer of photoreceptor cells. Photoreceptor cells are divided into cones and rods. The cell bodies of rods and cones form the outer nuclear layer. The photoreceptor forms synapses with the bipolar and horizontal cells that make up the outer plexiform layer. The cell bodies of bipolar cells, horizontal cells and amacrine cells form the inner nuclear layer. The synapses between retinal ganglion cells (RGCs), bipolar cells, and amacrine cells form the inner plexiform layer. The axons of RGC form the optic nerve, which connects the retina to the brain. The retinal neurovascular unit is composed of ganglion cells, glial cells (astrocytes and Müller cells), immune cells (microglia), and vascular cells (endothelial and pericytes), reflecting the complex functional coupling and interdependence between different retinal cells.

The retina's microvascular system is organized in a hierarchical fashion, with the deep vascular plexus on the outer surface of the INL, the intermediate vascular plexus on the border between the IPL and INL, and the superficial vascular plexus in the NFL.^50^ The term “neurovascular unit” was first used in the central nervous system and was later applied to the study of the retina. The neurovascular unit is an essential component of the normal retina, ensuring the integrity of the internal blood–retinal barrier (BRB) in response to the metabolic demands of retinal nerve cells^51^ (Figure 3). Directly in contact with the blood, endothelial cells form a semiselective barrier that regulates fluid and macromolecule flow between blood and the neuroretina via tight intercellular junctions.^52^ Pericytes control the morphology and function of blood vessels and frequently communicate with other cells in the NVU, collaborating to keep the BRB stable. Photoreceptors (cone and rod cells) in the retina communicate with secondary neurons (bipolar and horizontal cells), which communicate with ganglion cells. As the information‐integrating output neurons, ganglion cells’ axons form the NFL, which transmits electrical pulses converted from photoreceptors to the brain to form vision through the optic nerve.^53^

Most glial cells are Müller cells, which can be found in all retinal layers and in close contact with a wide variety of retinal cells. With their unique physiological structure, Müller cells can provide energy and a variety of neurotrophic factors to nerve cells, participate in the transport of neurotransmitters and ions, and are essential for the morphological stability of the BRB, protecting the integrity of neurons and the homeostasis of the retinal environment.^54^ Astrocytes are named for their star shape and are a type of glial cell that form irregular networks around neuronal axons and blood vessels to support the retina's integrity.^55^ Microglia have direct contact with neurons and pericytes and are found in the OPL, INL, IPL, GCL, and NFL of the retina under normal conditions. They maintain synaptic transmission and remove senescent damaged cells and metabolic waste products as highly specialized resident immune cells.^56^ Microglia support neuroretinal health via producing neurotrophic factors and anti‐inflammatory cytokines under physiological and acute inflammatory conditions. However, large amounts of proinflammatory cytokines are secreted in chronic retinal diseases to promote disease progression.^57^

### 4.2 Pathological changes of retinal NVU in DR

According to numerous studies, a high‐glucose environment causes significant damage to the NVU and retina.^58^ The pathological alterations of several representative cells in the NVU will be reviewed briefly.

Glial fibrillary acidic protein, a marker of Müller cell stress and gliosis, was found to be highly expressed in diabetes experimental models.^22^ Moreover, the high‐glucose environment decreases and displaces Kir4.1 channels on the cell surface^59^ while increasing the water channel protein Aqp4.^59^ These findings could be linked to retinal edema. In addition, neurotransmitter regulation is disrupted during DR disease. The current findings point to an increase in excitatory amino acid transporters EAAT1 and EAAT2, as well as in the uptake of glutamate by glucose‐deficient Müller cells. RGC protection by Müller cells may be enhanced at low glucose levels, and impaired glutamate uptake may result in retinal neurodegeneration.^60^ These findings show consistency with elevated glutamate levels in the retinas of DR animal models.^61^ Furthermore, a significant amount of VEGF is produced by Müller cells.^62^ Critical pathological features of DR, such as early vascular leakage and neovascularization, may involve Müller‐VEGF. Multiple pathways have been discovered to induce VEGF, and researchers discovered that Müller cells rely on VEGFR2 in an autocrine manner to determine VEGF upregulation.^63^ Loss of Müller cells and all other retinal nerve cells was the result of conditional VEGFR2 knockdown in Müller cells, which led to a significant reduction in BDNF and glial cell line‐derived neurotrophic factor.^64^ Furthermore, recent research has shown for the first time that in diabetic patients, VEGF upregulates BDNF production, highlighting the critical role of neuroprotection in Müller cells.^65^

Microglia are thought to be the first detectors of hyperglycemia signals in diabetics. Microglia undergo morphological changes from branched to amoeboid‐like; M1 cells are proinflammatory, and M2 cells are anti‐inflammatory.^66^ M1 phenotype activation typically increases the level of proinflammatory cytokines like TNF‐α, IL‐1β, IL‐6, and chemokines, as well as the production of cytotoxic mediators such as ROS and NO. Whereas M2 is characterized by its elevated expression of anti‐inflammatory cytokines.^67^ Microglia that are persistently activated produce an excess of cytokines, which can lead to neuroinflammation. At the same time, high glucose causes microglia to polarize to the M1 inflammatory phenotype, leading to an M1‐M2 imbalance. Recent research has discovered that injecting A20‐overexpression lentiviruses (OE‐A20) into the eye or oral administration of Asiatic acid can prevent diabetic rat retinal microglia from polarizing toward M1.^68^, ^69^ Cano‐Cano et al proposed that (Ss)‐DS‐ONJ pretreatment elevates the levels of heme oxygenase‐1 and IL‐10, thereby promoting M2 responses via arginase‐1 induction.^70^ The question of how to inhibit microglial M1 polarization or induce M2 polarization has become a hot topic, and changing the cell phenotype could be a prospective treatment for DR.

The basement membrane in the vessel wall is shared by endothelial cells (ECs) and pericytes, which allow for gap junctional communication at peg holes and other factors in paracrine signaling. TNF‐α and IL‐1 cytokines cause retinal endothelial cell dysfunction by inducing oxidative stress and activating signaling pathways like P38 MAPK and nuclear factor‐κB. Growth factors, on the other hand, such as transforming growth factor‐β increase endothelial cell permeability.^71^ Pericytes loss is also a key feature of DR, and dysregulation of pericyte‐EC communication exacerbates disease progression.^72^ The connection between ECs and pericytes is the subject of an increasing number of experiments. Jiang et al proposed a circRNA‐mediated mechanism in which cZNF532 induction or microRNA (miR)‐29a‐3p antagonism ameliorates pericyte degeneration caused by human diabetic vitreous and coordinates DR pericyte biology and intravascular homeostasis.^73^ In addition, vascular endothelial‐associated (VEAL2) lncRNA regulates junctional dynamics, maintains endothelial permeability, and reduces hyperpermeability, according to evidence from zebrafish and hyperglycemic human umbilical vein endothelial cell models, as well as patients with DR. Furthermore, treatments targeting cPWWP2A, miR‐579, and miR‐342‐3p all improved DR by modulating the survival of ECs and pericytes.^74^, ^75^

## 5 PATHOLOGICAL PROCESS OF AUTOPHAGY IN RETINAL NVU IN DR

### 5.1 Autophagy in Müller cells and astrocytes

Several studies have investigated autophagy in Müller cells, which are important glial cells in the retina. Researchers discovered autophagy induction in rat Müller cells after 48 h of high glucose treatment.^76^ Some authors, on the other hand, have suggested that Müller cells do not respond adequately to glucose‐induced stress and that autophagy is impaired due to lysosomal damage.^77^ Mecchia et al proposed that high glucose causes early activation and subsequent decline of autophagy in Müller cells.^78^ Therefore, the specific effects of glucose on autophagy must be investigated further.

**FIGURE 4:**
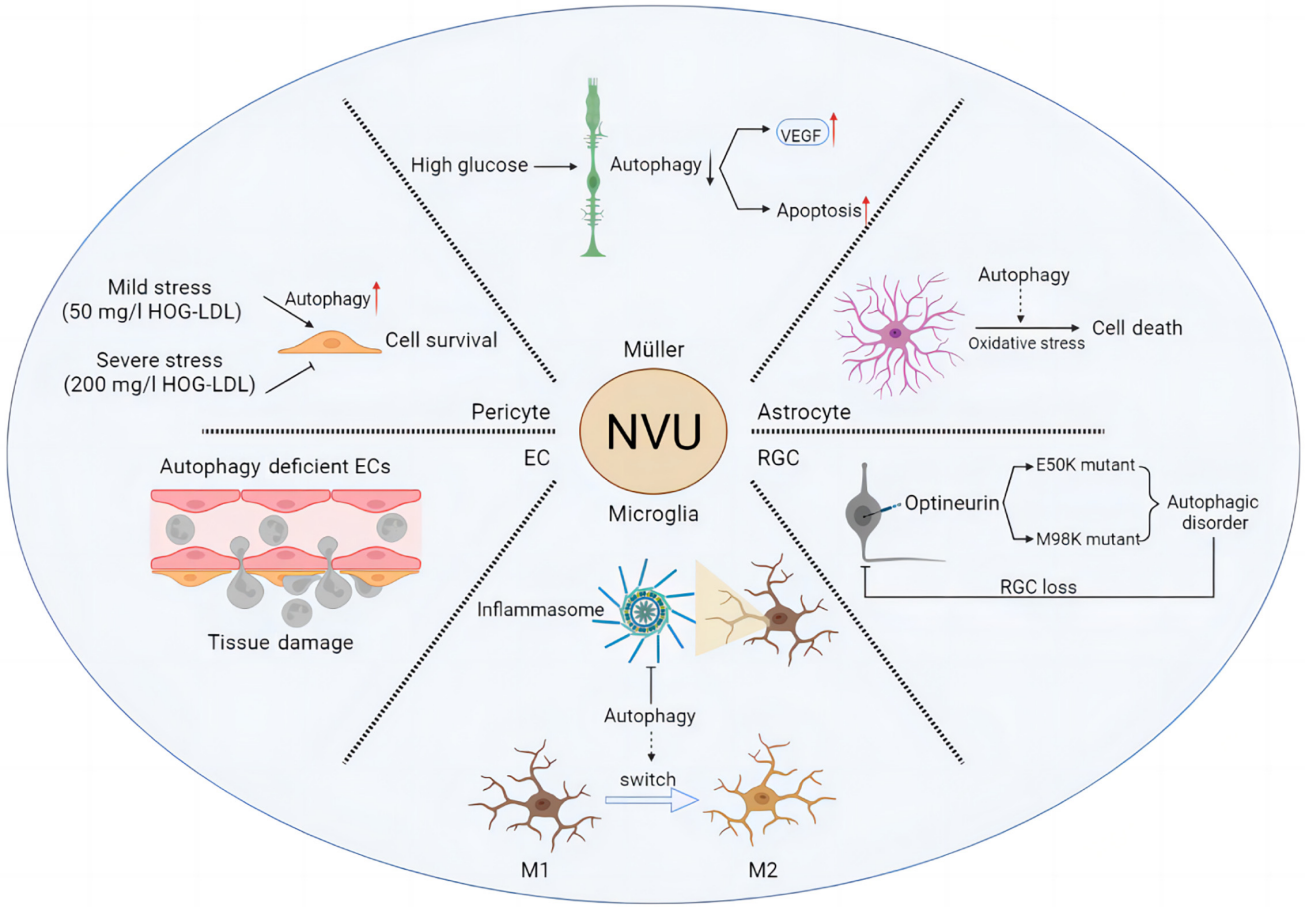
The pattern diagram of the representative studies of autophagy in different cells of NVU structure. EC, endothelial cell; HOG‐LDL, heavily oxidized glycated low‐density lipoprotein; NVU, neurovascular unit; VEGF, vascular endothelial growth factor.

Under high‐glucose conditions, oxidative stress causes an increase in misfolded or unfolded proteins, resulting in a sustained but insufficient endoplasmic reticulum stress response that may activate the autophagic machinery, producing more autophagosomes in the cytoplasmic lysosome and the accumulation of p62/SQTSM1 cargo. High glucose induces lysosome‐mediated autophagic dysfunction in DR rat Müller cells, resulting in increased apoptosis and the release of large amounts of VEGF (Figure 4). Rapamycin treatment prevents these changes.^79^ However, it has been discovered that defective autophagy makes Müller cells more vulnerable to oxidative stress^80^ and can kill cells via mitochondrial apoptosis, and that the sirtuin 4 (SIRT4)/AMPK axis, which is mediated by SIRT4, an acetylase in mitochondria, may be involved in regulating the autophagic process. According to new research, thioredoxin‐interacting protein (TXNIP) increases cell survival by selectively eliminating mitochondria through PTEN‐induced kinase 1 (PINK1)/Parkin‐mediated mitochondrial autophagy.^81^ TXNIP increased the expression of the nuclear protein HMGB1, which is transported from the nucleus under oxidative stress and interacts with Beclin‐1 to achieve autophagy in Müller cells. Thus, autophagy activation may represent a survival mechanism that degrades ROS‐damaged mitochondria.^76^ Furthermore, notoginsenoside R1,^82^ berberine,^83^ heparinase inhibitor PG545,^84^ and gypenoside XVII (Gyp‐17)^85^ have been shown to increase autophagy and thus ameliorate the lesions caused by DR via different pathways. According to these findings, maintaining Müller cells and retinal structure after oxidative damage requires adequate levels of autophagy.

Astrocytes are essential for maintaining the stability of the retina. Current research on astrocyte autophagy has primarily focused on neurological aspects, with little focus on the retina and DR. However, current research indicates that autophagy may influence astrocyte inflammation, oxidative stress, aging, and function.^82^ In vitro experiments have described potential mechanisms of autophagy regulation of oxidative stress in astrocytes. Autophagy has been shown to occur in astrocytes exposed to H_2_O_2_, and inhibiting autophagy significantly reduces H_2_O_2_‐induced cell death^86^ (Figure 4). According to these results, autophagy may play a role in oxidative stress‐induced astrocyte death. By disrupting and activating astrocytes, ROS can disrupt the homeostasis of the BRB and retinal environment via increasing the levels of cytokines and inflammatory mediators. Recently, Zhu et al discovered that astrocytes cause the onset and progression of retinal vascular diseases by releasing autophagy‐induced signals in the form of exosomes, which regulate the multiplication and migration of ECs in response to oxidative damage.^87^

### 5.2 Autophagy in retinal microglia

Neurodegenerative disorders like Parkinson's^88^ and Alzheimer's^89^ have led to extensive investigation into microglial autophagy. High glucose, on the other hand, induces microglial stress and activation,^90^ and microglia‐mediated inflammatory storms in DR are critical contributors to disease progression. Under normal conditions, autophagy inhibits the activity of inflammatory vesicles like NLRP3 in innate immune cells. He et al. discovered that when stimulated with lipopolysaccharide (LPS), unc‐51‐like kinase 1 (ULK1) phosphorylated by p38α MAPK in microglia becomes inactive and loses its ability to interact with ATG13, reducing autophagy flow and level. LPS‐induced inflammatory vesicle activity, IL‐1β production, and activation of microglial cell all require this inhibition. In contrast, blocking the initial signaling pathway activated by toll‐like receptor 4 (TLR4) was able to eliminate LPS's inhibitory effect on autophagy.^91^ Similarly, autophagic flux and Atg gene expression in microglia were significantly inhibited by TLR4 activation by LPS in the study by Lee et al.^92^ Based on these findings, the antidepressant fluoxetine significantly decreased LPS‐induced inflammatory cytokine and oxidative stress production in microglia while increasing phagocytosis and autophagy.^93^ In addition, diabetes‐induced neuroinflammation and microglial apoptosis can be prevented by administering melatonin, which activates autophagy in diabetic rats through the TLR4‐Akt–mTOR pathway.^94^ Despite increased expression of Tnfa and Il1β, Igf1 deficiency‐induced autophagy protected structure and function of the retina in 6‐month‐old mice due to reduced inflammatory vesicle activation in the retina.^95^ Furthermore, beta‐hydroxybutyrate reduced the expression of M1 microglia markers while increasing BDNF in the ONL of diabetic mice, reducing abnormal autophagy in microglia.^96^

As previously stated, artificially induced changes in microglial phenotype may become an important treatment avenue in the future. Antagonism of the peroxisome proliferator‐activated receptor γ (PPARγ) by the drug rosiglitazone appears to prevent LPS‐induced microglial activation. Reducing PPARγ levels stimulates autophagy in microglia and ends the M1‐M2 transition in these cells via the LKB1‐AMPK signaling pathway.^97^ Meanwhile, autophagy markers like LC3 and ATG7 were more widely expressed after dimethyl fumarate treatment, and in LPS‐activated microglia, dimethyl fumarate significantly suppressed NO and proinflammatory cytokine production through an autophagy‐dependent pathway and induced microglial cell transition to the M2 state.^98^ Cotreatment with the selective forkhead box protein O1 inhibitor AS1842856 or chloroquine suppressed autophagy in the brain's renin‐angiotensin system, blocking LPS‐induced NLRP3 inflammasome activation in response to Ang,^1^, ^2^, ^3^, ^4^, ^5^, ^6^, ^7^ and repolarizing microglia from M1 to M2^99^ (Figure 4). Finally, targeting the phenotypic shift of microglia is critical for DR blockade.

### 5.3 Autophagy in retinal ganglion cells

As the retina's only projection neurons, RGCs transmit visual information to the brain, and axons of RGCs form the optic nerve.^100^ Both retinal hypoxia and optic nerve axon injury have been shown to induce autophagy.^101^, ^102^ Researchers transduced RGCs with viral vectors targeting RGC serotypes, such as adeno‐associated virus (AAV2), to study the impact of downregulating autophagy in cells.^103^ ATG5FLOX/FLOX animals that received AAV2‐GFP‐CRE injections lived less than control mice. This was due to the downregulation of ATG5 in RGCs, which made cells more susceptible to optic nerve transection. These findings provide further evidence that autophagy protects RGCs from cell death after axonal injury. Optineurin (OPTN), a recently discovered autophagy receptor, is a glaucoma‐related protein that mediates the selective degradation of bacteria and mitochondria in the cells.^104^ Mutations in the OPTN gene, specifically E50K and M98K, are the most common inherited causes of normal tension glaucoma. The GTPase activating protein TBC1D17 is required for E50K‐OPTN‐induced autophagy, whereas the polymorphism of the glaucoma‐related optic nerve protein M98K causes autophagy, transferrin receptor degradation and retinal cell apoptosis.^105^ The E50K mutation significantly reduces retinal thickness and results in RGC loss in the peripheral retina in both transgenic mice and humans.^106^, ^107^ Based on these results, it appears that mutations of OPTN have a negative impact on retinal function and RGC survival in vivo (Figure 4). Furthermore, it has been discovered that succinic acid accumulates heavily in the retina of diabetic rats^108^ and that succinic acid accumulation can lead to RGC dysfunction in DR rats. Zhang et al discovered that in DR, RGCs are vulnerable to dysfunction of autophagy flow, but desuccinylation of the OPTN K108 site caused by Sirt5 provides protection.^109^

Although in the diabetic retina, the AMPK pathway has been shown to be activated, blocking this pathway has a modest effect on apoptosis of RGCs.^110^ However, phlorizin (PHL) treatment demonstrates that autophagy disorder caused by hyperglycemia results in neuronal loss and that neuronal protection in DR requires activation of the mTORC1 pathway. The percentage of apoptotic cells in the mouse GCL was lowered by PHL treatment, whereas injecting MHY1485, an mTOR activator to inhibit autophagy may provide greater protection to nerve cells.^111^ In terms of oxidative damage, trans‐reaction DNA binding protein 43 (TDP‐43) inhibition significantly inhibited H_2_O_2_‐induced apoptosis and autophagy. Reductions in the proapoptotic proteins such as Bax, cytochrome c, LC3II/I, and Beclin‐1 were accompanied by increased content of Bcl‐2, an antiapoptotic protein. TDP‐43 silencing reduced the expression of histone deacetylase 6 (HDAC6), and HDAC6 also eliminated TDP‐43's inhibitory effect on H_2_O_2_‐induced apoptosis and autophagy.^112^ It is worth noting that increased expression of LC3II/I and Beclin‐1 as well as p62 degradation, were observed in RGCs treated with lilarutide, which also inhibited autophagy. Furthermore, lilarutide can inhibit the decrease in GAP43 expression caused by H_2_O_2_, thereby protecting the cells. Rapamycin, on the other hand, can stop this process. By promoting mitochondrial production and decreasing mitochondrial autophagy, lilarutide can also alleviate the excessive production of ROS and decrease mitochondrial membrane potential brought on by H_2_O_2_. Finally, lilarutide can significantly reduce the decrease in cell survival rate, mitochondrial morphology, and autophagy caused by H_2_O_2_.^113^

### 5.4 Autophagy in pericytes and endothelial cells

Autophagy is a key regulator of many vascular diseases and can regulate EC survival and developmental angiogenesis.^114^, ^115^ Autophagy activation is the primary mechanism by which ECs respond to local disturbances such as hypoxia, infection, and trauma.^116^ The incidence and course of inflammation are intimately linked to ECs. Recent research has discovered that autophagy can control the remodeling of connections as well as the expression of key adhesion molecules. In many inflammatory models, EC autophagy is impaired, resulting in excessive neutrophil infiltration. Therefore, autophagy is a critical cellular process that regulates physiological neutrophil trafficking.^117^ More study has indicated that EC autophagy impairment can lead to a rise in proinflammatory cytokines, IL‐6‐dependent endothelial‐mesenchymal transition, and organ fibrosis associated with metabolic abnormalities^118^ (Figure 4). These findings imply that EC autophagy has a distinct role in vascular biology.

In addition, mitochondrial dysfunction contributes significantly to EC dysfunction. Apoptosis can be avoided in high glucose‐induced ECs by preventing mitochondrial rupture, signaling that modulation of mitochondrial dynamics could be a viable target for damage treatment. Rapamycin increased ROS levels and apoptosis in rats in a high glucose environment by activating mitochondrial autophagy, whereas dynamic protein‐related protein 1(Drp1) knockout inhibited autophagy flux and decreased LC3‐II expression.^119^ Interestingly, Zhang et al discovered that high glucose not only induced mitochondrial excessive division and production of damaged mitochondria by promoting PKCδ/Drp1 signal transduction, but it also inhibited mitochondrial autophagy by inhibiting LC3B‐II formation and p62 degradation, as well as limited clearance of damaged mitochondria.^120^ Similar findings have been confirmed in a study of the TGR5 signal's protective effect on DR.^121^ These findings imply that maintaining mitochondrial homeostasis is critical for ECs and that inhibiting Drp1 may be the key to treating DR.

Furthermore, Sirt3 expression has been linked to DR and has been found to be negatively correlated with the duration of the lesion. Sirt3 overexpression has been shown in bovine and diabetic rats to protect retinal ECs from hyperglycemia‐induced damage.^122^ Sirt3 inhibited matrix metalloproteinase‐2, VEGF, HIF‐1, and insulin‐like growth factor‐1 expression while increasing the mRNA content of LC3 and the protein content of LC3‐II.^123^, ^124^ Sirt3 may be a potential medication for the treatment of retinal neovascularization‐related illnesses by controlling the expression of migration, neovascularization, and autophagy‐related proteins.

Pericytes are required for the construction and function of retinal capillaries, and their loss is a symptom of DR. According to certain research, diabetic individuals’ retinas have exosmosis and DR oxidation that is related to the severity of low‐density lipoprotein (LDL).^125^ Pericyte loss enhances BRB permeability, which aids LDL penetration into the retina. Pericytes exposed to a sublethal dosage of LDL showed enhanced autophagy, protecting cell survival. At high LDL levels, however, this prosurvival autophagy is no longer evident, and autophagy suppression via Beclin‐1 can sustain pericyte survival^8^ (Figure 4). Endoplasmic reticulum stress is a key cause of pericyte loss and retinal damage in DR.

Unfolded protein response can overcome moderate endoplasmic reticulum stress, but excessive and long‐term endoplasmic reticulum stress can lead to apoptosis.^126^ The DR rat model also exhibits this dual effect of autophagy induced by endoplasmic reticulum stress.^8^

## 6 DISCUSSION

Blindness is one of the most incapacitating disabilities, according to the World Health Organization. As a result, more difficult‐to‐cure and irreversible diseases, such as DR, have emerged as important targets for prevention and treatment. Many people are looking for effective pathology treatment in order to save their DR vision.

There is emerging evidence that the retinal NVU serves as the functional foundation of the retina, and that NVU damage can result in aberrant physiological function and even retinopathy. The studies in this review emphasize the importance of autophagy in maintaining retinal NVU structure and function in DR. Autophagy is triggered in the majority of retinal NVU cells in diabetics. Autophagy, on the other hand, is a two‐edged sword; whereas moderate autophagy can help retinal NVU cells resist the effects of ROS, inflammation, and trauma, excessive autophagy levels can cause abnormal cell death. Autophagy maintains mitochondrial homeostasis in a variety of cells, including Müller and ECs, and removes damaged mitochondria, preventing accumulation. Autophagy in microglia may be a novel way to regulate cell phenotype and thus prevent the cytokine storm induced by microglia. Autophagy also protects RGCs from axonal damage and oxidative stress, resulting in decreased cell loss. Additionally, autophagy controls the expression of vital ECs adhesion molecules, the remodeling of ECs junctions, and its function in controlling neovascularization. As a result of the review, changes in the autophagy‐lysosome pathway are linked to the development of DR. Future studies will likely help us better comprehend the possibilities of therapeutic approaches that target it in DR.

However, there are numerous challenges. One of these difficulties stems from the tissue's complexity. Different types of cells in the NVU are primarily affected by DR, and these cells may respond very differently under pathological conditions. Furthermore, macroautophagy may occur in conjunction with other degradation pathways. For example, in AMD, there was a significant decrease in retinal macroautophagy activity that was accompanied by an increase in CMA activity, which compensated for the decrease in macroautophagy.^20^ These synergistic effects could lead to new treatment opinions.

Drug therapy, laser photocoagulation and surgery are the three major therapy methods for DR. Pharmacotherapy intervenes in multiple aspects of DR progression while better preserving the retinal anatomy. Therefore, understanding the role of autophagy in multiple aspects of DR is beneficial in targeting the prevention and slowing the progression of the disease. However, on the underlying molecular mechanisms and potential functions of autophagy in retinal NVU cells, there have been few published investigations. As a result, more study is required to determine the fundamental function of autophagy in DR.

## 7 CONCLUSIONS

Under the influence of DR, each structure of NVU shows different pathological changes, and autophagy also plays a variety of roles in it. Undoubtedly, more and more studies have found that autophagy therapy targeting NVU cells may be extremely important for blocking DR progression. We discovered many potential therapeutic targets by elucidating the molecular mechanism of different cells involved in DR via autophagy. However, more research is needed to investigate this complex mechanism, as well as more mechanisms of reference significance for drug therapy.

## AUTHOR CONTRIBUTIONS

Xiongyi Yang conceived of and developed the structural design of the article and wrote the manuscript. Zexin Huang organized the relevant literature, wrote the manuscript, and made important revisions. Mei Xu, Yanxia Chen, Mingzhe Cao, Guoguo Yi, and Min Fu improved the layout and structure of the paper and provided suggestions for important revisions. All the authors reviewed and edited the manuscript and agreed to the published version of the manuscript.

## DISCLOSURE

Xiongyi Yang, Zexin Huang, Mei Xu, Yanxia Chen, Mingzhe Cao, Guoguo Yi, and Min Fu declare that they have no competing interests. No human or animal studies involved or no ethical statement for the study. No data were used for the research described in the article.
